# Structure-guided design and cloning of peptide inhibitors targeting CDK9/cyclin T1 protein-protein interaction

**DOI:** 10.3389/fphar.2024.1327820

**Published:** 2024-05-14

**Authors:** Mohammad Sadegh Taghizadeh, Mohsen Taherishirazi, Ali Niazi, Alireza Afsharifar, Ali Moghadam

**Affiliations:** ^1^ Institute of Biotechnology, Shiraz University, Shiraz, Iran; ^2^ Plant Virology Research Center, School of Agriculture, Shiraz University, Shiraz, Iran

**Keywords:** drug discovery, MMPBSA, molecular docking, molecular dynamics simulation, mutagenesis, peptide inhibitor

## Abstract

CDK9 (cyclin-dependent kinase 9) plays a significant role in numerous pathological conditions, such as HIV-1 infection and cancer. The interaction between CDK9 and cyclin T1 is crucial for maintaining the kinase’s active state. Therefore, targeting this protein-protein interaction offers a promising strategy for inhibiting CDK9. In this study, we aimed to design and characterize a library of mutant peptides based on the binding region of cyclin T1 to CDK9. Using Osprey software, a total of 7,776 mutant peptides were generated. After conducting a comprehensive analysis, three peptides, namely, mp3 (RAADVEGQRKRRE), mp20 (RAATVEGQRKRRE), and mp29 (RAADVEGQDKRRE), were identified as promising inhibitors that possess the ability to bind to CDK9 with high affinity and exhibit low free binding energy. These peptides exhibited favorable safety profiles and displayed promising dynamic behaviors. Notably, our findings revealed that the mp3 and mp29 peptides interacted with a conserved sequence in CDK9 (residues 60–66). In addition, by designing the structure of potential peptides in the plasmid vector pET28a (+), we have been able to pave the way for facilitating the process of their recombinant production in an *Escherichia coli* expression system in future studies. Predictions indicated good solubility upon overexpression, further supporting their potential for downstream applications. While these results demonstrate the promise of the designed peptides as blockers of CDK9 with high affinity, additional experimental studies are required to validate their biological activity and assess their selectivity. Such investigations will provide valuable insights into their therapeutic potential and pave the way for the future development of peptide-based inhibitors targeting the CDK9-cyclin T1 complex.

## Introduction

The importance of battling cancer cannot be overstated, given its devastating effects on numerous individuals, including substantial morbidity and mortality rates worldwide. Thus, utilizing the knowledge gained from prior research and breakthroughs in biological sciences, it is crucial to develop a scientifically sound and effective treatment strategy for pathological diseases, including cancer. There are two isoforms of cyclin-dependent kinase 9 (CDK9) in humans that differ in their N-terminus structure (Shore et al., 2003), and when these isoforms interact with cyclins T1, T2, and K, they form the P-TEFb (positive transcription elongation factor b) protein complex, which plays a vital role in elongating RNA polymerase II transcripts (Peng et al., 1998). According to reports, the P-TEFb complex has a notable involvement in various pathological conditions, such as HIV-1 infection, cardiac hypertrophy, some types of cancers, and chronic inflammatory conditions (Romano and Giordano, 2008; Wang and Fischer, 2008; Kryštof et al., 2010; Krystof et al., 2012). Moreover, monomeric CDK9 is unstable following synthesis within the cell and requires the support of cytoplasmic proteins including Cdc37, Hsp70, and Hsp90 to form a stable complex with its cyclin partner (O'Keeffe et al., 2000). Hence, a potential strategy for inhibiting the involvement of CDK9 in the above pathological conditions, especially apoptosis and angiogenesis of cancers (Kryštof et al., 2011; Polier et al., 2011), is to disrupt its binding to its cyclin partner.

Flavopiridol and other small molecules have been identified as powerful inhibitors of CDK9. However, they may also inhibit other CDKs and protein kinases due to their competition with the highly conserved catalytic ATP binding site (Sonawane et al., 2016). This limits their potential as therapeutic agents or chemical probes (Asghar et al., 2015). Furthermore, inhibitors face the challenge of binding to high intracellular levels of ATP and, although some progress has been made in improving the selectivity of CDK9 inhibitors by utilizing the conformational changes of the glycine-rich loop of CDK9 (Baumli et al., 2010), there is still a need for compounds with even greater selectivity. Although targeting protein-protein interactions for drug development is challenging, several inhibitors of such interactions have been achieved with success, with some even undergoing clinical trials (Gandhi et al., 2011) or being approved for therapeutic use (Kuritzkes et al., 2008). On the other hand, using peptides as an alternative approach is advantageous because they can occupy a distinct chemical space and interact with proteins that are challenging to target with small molecules (Taghizadeh et al., 2022b). Moreover, peptides have shown a higher degree of specificity towards their target proteins, resulting in increased efficacy and fewer off-target effects (Cunningham et al., 2017). Additionally, peptides can be chemically modified to improve their stability and are easily synthesized, making them a promising tool for developing alternative therapeutics that target protein-protein interactions (Cunningham et al., 2017). To date, only one study has successfully developed direct inhibitors targeting the CDK9-cyclin T1 interaction, which involved the design of ten native peptides, ranging from 4 to 8 residues, based on fragments extracted from either CDK9 or cyclin T1 (Randjelović et al., 2013).

The aim of this study was to develop cell-penetrating peptides that can target the CDK9-cyclin T1 complex and prevent its assembly. We designed three distinct peptide templates using the cyclin T1 binding site of CDK9 and evaluated their interactions and stability in binding to the CDK9. One peptide template was then selected for further optimization of its effectiveness through mutagenesis at specific positions to modify the binding energy and occupancy percentage to the CDK9. The OSPREY (Open-Source Protein Redesign for You) tool was employed for this purpose. We evaluated the changes in binding energy and stability, along with the drug-like properties of the peptides. As a result, we identified three promising 13-amino acid peptides that could effectively target the CDK9-cyclin T1 complex. Expression constructions were designed to facilitate recombinant production in *Escherichia coli* cells for the future studies. Our findings may lead to the development of drugs targeting the CDK9-cyclin T1 complex in the future.

## Materials and methods

### CDK9-cyclin T1 interface analysis and template extraction

The crystal structure of the CDK9-Cyclin T1 complex, as represented by the PDB ID: 6Z45, was retrieved from the RCSB protein data bank (https://www.rcsb.org/). Subsequently, the LigPlot^+^ v. 2.2.8 software (European Bioinformatics Institute, Hinxton, UK) (Laskowski and Swindells, 2011) was used to identify and visualize the interactions occurring at the interface between the cyclin T1 and the CDK9. Through interface analysis, three peptide templates were extracted using the UCSF Chimera v.1.14 software (University of California, San Francisco, United States) (Pettersen et al., 2004), namely, temp1 (residues 70–80), temp2 (residues 90–102), and temp3 (residues 136–148). This process was guided by the understanding that cell-penetrating peptides are typically composed of 5–30 amino acids in length (Borrelli et al., 2018). Afterward, the resolving of the missing atoms in the CDK9 protein extracted from the complex was conducted using MODELLER provided by the ModLoop server (https://modbase.compbio.ucsf.edu/modloop/). In addition, the protein and peptides were refined for molecular docking by adding hydrogen and charge, and removing interacting ligands and water molecules using UCSF Chimera.

### Molecular docking analysis

The HADDOCK 2.4 server (https://wenmr.science.uu.nl/haddock2.4/) with default parameters was used to perform molecular docking analysis. The binding site of the CDK9 protein was determined to be the site of interaction with cyclin-T1, and residues Asp7, Glu9, Cys10, Glu57, Phe59, Arg65, Gln71, and Arg86 of the CDK9 protein (Barlaam et al., 2020), along with all residues of the peptides, were selected as binding sites. The complex with the lowest binding scores, temp2, was chosen for further analysis. The same methodology was used for subsequent docking analyses, and all complexes were visualized using the BIOVIA Discovery Studio v.20.1.0 (Dassault Systèmes BIOVIA, San Diego, United States).

### Molecular dynamics (MD) simulation

The dynamic behaviors of the potentially effective mutant peptides or template, the strength of their interaction with the receptor, and the overall stability of the complexes were investigated through MD simulations using the GROMACS software package v.2022 (University of Groningen, Groningen, Netherlands) (Van Der Spoel et al., 2005). The topology files were then generated using the Gromos96 54a7 forcefield due to its ability to exhibit good agreement with both the X-ray crystal structure and NMR data of the protein (Stocker and van Gunsteren, 2000). The complexes were then solvated using the simple-point charge (SPC) water model and neutralized by the addition of NA or CL ions. The resulting systems were energy minimized with the steepest descent method over 50,000 steps and equilibrated under a pressure of 1 bar and at a temperature of 310.15 K. Finally, a 100 ns molecular dynamics simulation was run using GROMACS on GPU with the equilibrated complexes.

### MD trajectories analysis

The GROMACS software tools were used to analyze various structural properties of the MD trajectories, such as root mean square deviation (RMSD), root mean square fluctuation (RMSF), surface accessible solvent area (SASA), and radius of gyration (Rg). The *gmx hbond* toolkit and readhbmap. py python script was used to respectively evaluate the number and occupancy percentage of hydrogen bonds. Principal component analysis (PCA) was applied during the last 10 ns of simulations to identify the dominant and collective motions of the protein, with the covariance matrix calculated using the *gmx covar* tool. The covariance matrix was diagonalized to obtain eigenvalues and eigenvectors, and the *gmx anaeig* tool was used to provide a two-dimensional plot for the PCA. To obtain the projection plots over time, we utilized the *gmx anaeig* tool with -proj flag, which calculates the inner product of the configuration (coordinates) with the eigenvector. In addition, the entropy of CDK9 and the associated complexes was computed using Schlitter’s method, utilizing the *gmx covar* tool and subsequently the *gmx anaeig* tool (Schlitter and Massarczyk, 2019). The *gmx mdmat* tool was also employed to generate distance matrices that observed interactions between mutated peptides and CDK9 by comprising the minimum distance between pairs of residues.

The total free binding, polar solvation, SASA, electrostatic, and van der Waals energies of each residue of temp2, temp2, and mutant peptides were obtained using the g_mmpbsa program (Kumari et al., 2014). Prior to conducting the analysis on our complexes, we optimized the analysis by utilizing two pre-designed peptide sequences developed by Randjelović et al. (2013). These sequences, namely, LQTLGFEL (representing cyclin T1 residues 141–148 as the positive control) and AAAAA (serving as the negative control), were employed in complex with CDK9 for the purpose of the optimization process. Furthermore, the *gmx do_dssp* analysis was carried out to compare the secondary structure frequencies in CDK9 complexed with peptides (Frishman and Argos, 1995).

### Peptide library construction

Before constructing the library, we calculated the binding energy and occupancy percentage of each residue in temp2. Residues with positive energy and low occupancy were selected for substitution with desired residues such as Arg, Asp, Ser, Thr, Trp, and Ile. The selection was based on their flexibility and probability, which were determined using the setLibraryRotamers scripts. The Open-Source Protein Redesign for You (OSPREY v.3.3; Duke University, North Carolina, United States) Python script was used to construct the library (Hallen et al., 2018). The script was executed with default parameters, following the OSPREY documentation. We also improved the script to allow output categorization in an excel file (code.py in the Supplementary Material S1).

### Evaluation of drug-likeness properties

The drug-like properties of the Top-30 mutant peptides, such as allergenicity, toxicity, and intestinal stability, were assessed using the AllerTOP v.2.0 server (http://www.ddg-pharmfac.net/AllerTOP/), ToxinPred server (https://webs.iiitd.edu.in/raghava/toxinpred/protein.php), and HLP server (http://crdd.osdd.net/cgibin/hlp/interactive.pl), respectively, with default parameters. Furthermore, the cell-penetrating ability of peptides was evaluated using the CellPPD server (http://crdd.osdd.net/raghava/cellppd/).

### Codon optimization and *in silico* cloning

Codon adaptation involves optimizing the codons in a foreign gene to match those present in the host gene, thereby improving gene expression quality (Nain et al., 2020). To achieve this, we utilized the JCat server (http://www.jcat.de/), a Java Codon Adaptation Tool, to optimize the codons of our potential effective peptides, including mp3, mp20, and mp29, according to the widely used *E. coli* strain K12. To ensure conservation in prokaryotic systems, we avoided three additional options offered by the JCat server (Sharp and Li, 1987). Furthermore, we integrated *NdeI* and *XhoI* restriction sites at the C and N terminals of the optimized DNA sequences, respectively. Subsequently, we employed the SnapGene v7.0.2 tool (GSL Biotech (LLC), Chicago, United States) to separately clone the optimized DNA sequences into the *E. coli* pET28a (+) vector for recombinant production in the future.

In addition, the solubility of the recombinant three selected peptides upon overexpression in *E. coli* was predicted using the SOLpro tool (https://scratch.proteomics.ics.uci.edu/). This analysis was conducted as a preliminary step towards subsequent structural, functional, and biochemical investigations.

## Results and discussion

### Template extraction and computational peptide design

**FIGURE 1:**
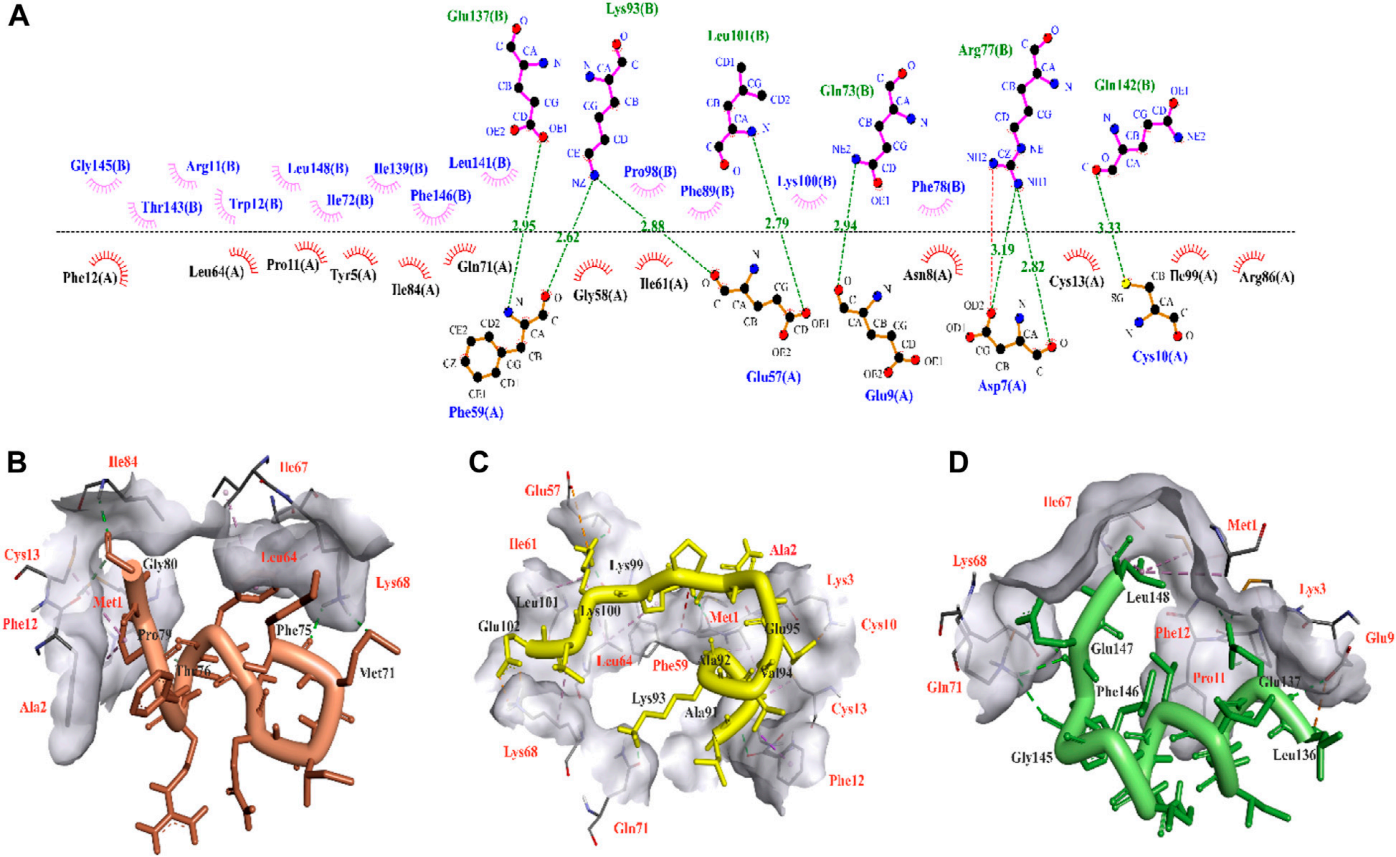
3D illustration of CDK9-cycline T1 and molecular interaction modes of three extracted templates. **(A)** CDK9-Cyclin T1 interface analysis using LigPlot for extracting three potential templates, in which chain A and chain B are related to CDK9 and Cyclin T1 residues, respectively. Hydrogen bonds are shown in green dash lines with respect to their distances. **(B)**, **(C)**, and **(D)** respectively show molecular interaction modes of temp1, temp2, and temp3 in complex with CDK9 protein. The interacted residues of CDK9 were indicated in red color.

Protein kinases, the largest class of pharmacological targets after G protein-coupled receptors (Bhullar et al., 2018), play a significant role in various pathological conditions, particularly in the context of cancer initiation and progression. CDK9, through its interaction with cyclin T1 and the formation of the P-TEFb complex, exerts a significant impact on the development of these diseases (Kryštof et al., 2011; Polier et al., 2011). Hence, in this study, the analysis of the interface of a previous experimentally reported CDK9-cyclin T1 complex (PDB ID: 6Z45) was employed to identify the specific residues engaged in their interaction. By conducting the analysis, it was determined that cyclin T1 residues Glu73, Arg77, Lys93, Leu101, Glu137, and Gln142 have the capability to form hydrogen bonds with CDK9 residues Asp7, Glu9, Cys10, Glu57, and Phe59 (Figure 1A). This interaction analysis sheds light on the key molecular interactions that play a crucial role in the development of potent peptide inhibitors. Taking into account the ability of the designed peptides to effectively enter cells due to their size (5–30 amino acids) (Borrelli et al., 2018), this study extracted three templates from the binding region of cyclin T1 to CDK9. The molecular docking analysis was conducted utilizing the HADDOCK server to validate binding potential of templates, revealing that temp2 exhibited superior interaction capability with a HADDOCK score of −78.7 and a Z-score of −2.8 compared to temp1 (HADDOCK score of −68.5 and a Z-score of −1.6) and temp3 (HADDOCK score of −75.1 and a Z-score of −2.1). As previously mentioned, the active site of CDK9 comprises Asp7, Glu9, Cys10, Glu57, Phe59, Arg65, Gln71, and Arg86, which serve as the binding sites for cyclin T1 (Barlaam et al., 2020). The molecular docking results for temp1 revealed that residues Met71, Phe75, Thr76, Pro79, and Gly80 interacted with residues Met1, Ala2, Phe12, Cys13, Leu64, Ile67, Lys68, and Ile84 of CDK9 (Figure 1B), while residues Ala91, Ala92, Lys93, Val94, Glu95, Lys99, Lys100, Leu101, and Glu102 of temp2 demonstrate the ability to establish bonding interactions with residues Met1, Ala2, Lys3, Cys10, Phe12, Cys13, Glu57, Phe59, Ile61, Leu64, Lys68, and Gln71 of CDK9 (Figure 1C). Furthermore, in the case of temp3, residues Leu136, Glu137, Gly145, Phe146, Glu147, and Leu148 were found to interact with residues Ala2, Glu9, Cys10, Pro11, Phe12, Cys13, Lys68, Gln71, and Ile84 of CDK9, as depicted in Figure 1D. Within CDK9, there exists a highly conserved peptide sequence, PITALRE (residues 60–66), that plays a crucial role in the interaction with cyclin T1 during the activation of CDK9 (Baumli et al., 2008). In summary, the results demonstrate that temp2 serves as a more effective barrier against cyclin T1 binding to CDK9 compared to the other two templates. Consequently, temp2 was selected as the preferred template for generating mutations, aiming to produce peptides with enhanced efficacy. In designing the peptides, two essential criteria were taken into logical consideration, (i) the peptide inhibitors should exhibit a higher affinity for CDK9 than temp2 to effectively compete with cyclin T1, and (ii) the peptide inhibitors exhibit cell-penetrating ability without inducing cytotoxicity or allergenicity. It is important to note that these predictions must be verified through experimental studies to evaluate their effectiveness in enabling CDK9 to regulate gene expression and cellular processes in normal cells (Falco and Giordano, 2002; Bhullar et al., 2018; Franco et al., 2018).

Given that the binding energy in protein-protein interactions is primarily influenced by a small number of crucial hot-spot amino acids (Bogan and Thorn, 1998), we employed g_mmpbsa and H-bond occupancy analyses to identify unfavorable residues for mutation, as they make significant contributions to the overall binding energy. To identify regions of relatively weak interactions between the temp2-CDK9 complex that could be potentially replaced by alternative amino acids in the sequence, a 100 ns molecular dynamics (MD) simulation was performed. The utilization of g_mmpbsa analysis revealed that certain residues, namely, Leu90, Lys93, Val94, Gln97, Lys99, and Lys100, exhibited unfavorable binding energies, indicating their detrimental impact on the binding of temp2 to CDK9 (Table 1).

**TABLE 1:** Determining residue binding energy in temp2 following a 100 ns molecular dynamics simulation using g_mmpbsa analysis.

Residues	Binding energy (kJ/mol)
Leu90	115.019
Ala91	−4.432
Ala92	−1.913
Lys93	120.953
Val94	22.362
Glu95	−147.885
Gly96	−15.642
Gln97	0.714
Pro98	−12.362
Lys99	133.148
Lys100	154.817
Leu101	−4.239
Glu102	−311.340

On the other hand, considering the essential role of hydrogen bonds in protein-protein interactions (Zareei et al., 2022), we performed an analysis of H-bond occupancy to investigate their involvement in the present study. The results indicated that residues Val94, Gly96, Gln97, and Lys99 exhibited the highest occupancy percentage for H-bond formation, whereas residues Pro98, Lys100, and Leu101 were observed to be incapable of forming H-bonds (Table 2). Based on the comparison of the two results, residues Leu90, Lys93, Pro98, Lys100, and Leu101 of temp2 were chosen to be substituted with alternative amino acids using the Osprey software. As a result, a library comprising 7,776 mutant peptides was generated (refer to the Supplementary Material S2), which was utilized for the assessment of effective peptides. To the best of our knowledge, the design and development of a mutant peptide library targeting the CDK9-cyclin T1 complex has not been undertaken thus far using the same methodology. However, previous studies have employed phage biopanning technique and alanine scanning analysis to design mutant peptide inhibitors against cancer targets (Saw and Song, 2019; Shahab et al., 2023). Moreover, a similar methodology has been employed to design effective mutant peptides that inhibit the interaction between the spike protein of SARS-CoV-2 and the human ACE2 receptor (Pourmand et al., 2022).

**TABLE 2:** Analysis of hydrogen bond occupancy between temp2 and CDK9 protein.

Pair ID	Donor-acceptor	Atom number	Occupancy (%)
1	**Lys99** (H)–Phe59 (O)	3,497–627	82.6
2	**Gln97** (E21)–Glu57 (OE2)	3,485–603	30.4
3	**Gln97** (E21)–Glu57 (OE1)	3,485–602	27.2
4	**Gln97** (H)–Glu57 (O)	3,478–605	29.1
5	**Gly96** (H)–Met1 (O)	3,473–11	80.6
6	**Ala92** (H)–Glu9 (OE2)	3,436–97	24.4
7	**Ala92** (H)–Glu9 (OE1)	3,436–96	19.1
8	**Ala91** (H)–Glu9 (OE2)	3,430–97	24.0
9	**Ala91** (H)–Glu9 (OE1)	3,430–96	25.6
10	Thr62 (HG1)–**Glu102** (OE2)	649–3,538	14.6
11	Thr62 (HG1)–**Glu102** (OE1)	649–3,537	13.7
12	Thr62 (H)–**Glu102** (OE2)	645–3,538	11.3
13	Thr62 (H)–**Glu102** (OE1)	645–3,537	11.2
14	Ile61 (H)–**Lys99** (O)	636–3,508	29.0
15	Phe59 (H)–**Gln97** (O)	612–3,488	60.0
16	Glu9 (H)–**Leu90** (O)	91–3,428	11.4
17	Asn8 (D21)–**Lys93** (O)	86–3,453	11.7
18	Lys3 (H)–**Val94** (O)	19–3,461	75.8
19	Met1 (H1)–**Glu95** (OE2)	2–3,469	13.4
20	Met1 (H1)–**Glu95** (OE1)	2–3,468	13.5

***Note:*** The residues of temp2 were highlighted in Bold.

### Exploring the potential effective peptide inhibitors of the CDK9-cyclin T1 complex

As previously highlighted, one of our objectives was to design safe peptides that possess a non-toxic nature. Furthermore, to ensure the effectiveness of bioactive peptides, it is crucial for them to possess resistance against gastrointestinal digestion, allowing for an optimal retention time within the body (Pei et al., 2022). On the other hand, allergenic peptides have the potential to elicit immune responses and trigger allergies, leading to a reduction in their demand (Shriver and Yang, 2011). Despite being aware of these considerations, a subset of 30 peptides with the highest Osprey scores was selected from the generated peptide library for evaluating their allergenicity, toxicity, stability half-life under intestinal conditions, and cell-penetrating ability. The results showed that none of the mutant peptides exhibited toxicity (Table 3). Among the 30 mutant peptides, 8 peptides displayed allergenicity, while 6 peptides demonstrated high intestinal stability (Table 3). Moreover, each of them demonstrated the capacity to penetrate cells (Table 3). Consequently, the selection process resulted in six peptides− mp3, mp7, mp12, mp20, mp26, and mp29− chosen as effective inhibitors of the CDK9-cyclin T1 complex due to their non-toxic, non-allergenic, high intestinal stability properties, and cell-penetrating capacity.

**TABLE 3:** Assessing drug-likeness properties of Top-30 mutant peptides ranked by OSPREY scores.

ID	Sequence	OSPREY score	Allergenicity	Toxicity	Intestinal stability (half-life (sec.))	Cell-penetrating capacity
mp1	R AA R VEGQ R K RR E	−109.923	NO	Non-Toxin	Normal (0.581)	CPP
mp2	D AA R VEGQ R K RR E	−103.587	NO	Non-Toxin	Normal (0.580)	CPP
mp3	R AA D VEGQ R K RR E	−103.429	NO	Non-Toxin	High (1.439)	CPP
mp4	R AA R VEGQ R K RD E	−101.902	NO	Non-Toxin	Normal (0.689)	CPP
mp5	R AA R VEGQ R K DR E	−101.353	YES	Non-Toxin	Normal (0.473)	CPP
mp6	R AA R VEGQ D K RR E	−99.400	YES	Non-Toxin	Normal (0.570)	CPP
mp7	R AA W VEGQ R K RR E	−95.470	NO	Non-Toxin	High (1.119)	CPP
mp8	R AA R VEGQ W K RR E	−94.064	YES	Non-Toxin	Normal (0.650)	CPP
mp9	D AA R VEGQ R K DR E	−93.781	YES	Non-Toxin	Normal (0.472)	CPP
mp10	W AA R VEGQ R K RR E	−93.673	NO	Non-Toxin	Normal (0.631)	CPP
mp11	R AA R VEGQ R K RW E	−93.571	NO	Non-Toxin	Normal (0.749)	CPP
mp12	R AA D VEGQ R K DR E	−93.464	NO	Non-Toxin	High (1.331)	CPP
mp13	R AA S VEGQ R K RR E	−93.450	NO	Non-Toxin	Normal (0.618)	CPP
mp14	R AA R VEGQ R K RT E	−93.246	NO	Non-Toxin	Normal (0.983)	CPP
mp15	R AA R VEGQ R K RS E	−93.204	NO	Non-Toxin	Normal (0.736)	CPP
mp16	R AA R VEGQ R K TR E	−93.158	NO	Non-Toxin	Normal (0.438)	CPP
mp17	T AA R VEGQ R K RR E	−93.099	NO	Non-Toxin	Normal (0.643)	CPP
mp18	R AA R VEGQ R K WR E	−93.012	YES	Non-Toxin	Normal (0.581)	CPP
mp19	S AA R VEGQ R K RR E	−92.897	NO	Non-Toxin	Normal (0.593)	CPP
mp20	R AA R VEGQ R K RR E	−92.859	NO	Non-Toxin	High (1.039)	CPP
mp21	R AA R VEGQ R K SR E	−92.489	NO	Non-Toxin	Normal (0.560)	CPP
mp22	D AA R VEGQ R K RD E	−92.200	NO	Non-Toxin	Normal (0.688)	CPP
mp23	R AA R VEGQ T K RR E	−91.595	NO	Non-Toxin	Normal (0.745)	CPP
mp24	D AA R VEGQ D K RR E	−91.360	YES	Non-Toxin	Normal (0.569)	CPP
mp25	R AA R VEGQ S K RR E	−90.742	NO	Non-Toxin	Normal (0.737)	CPP
mp26	D AA D VEGQ R K RR E	−90.152	NO	Non-Toxin	High (1.448)	CPP
mp27	R AA R VEGQ R K DD E	−90.142	YES	Non-Toxin	Normal (0.714)	CPP
mp28	R AA R VEGQ D K DR E	−89.865	NO	Non-Toxin	Normal (0.462)	CPP
mp29	R AA D VEGQ D K RR E	−89.746	NO	Non-Toxin	High (1.428)	CPP
mp30	R AA R VEGQ D K RD E	−88.732	YES	Non-Toxin	Normal (0.678)	CPP

***Note:*** mutated positions were highlighted in red color. CPP: cell-penetrating peptide.

Upon selecting the effective inhibitors, we conducted molecular docking and molecular dynamics analyses to screen and identify mutant peptides with a higher binding affinity and the lowest binding energy to CDK9, aiming to pinpoint potentially effective candidates. During activation of CDK9, the phosphorylation of Thr186 facilitates the formation of an intramolecular hydrogen bonding network involving Arg148 and Arg172, leading to the complete activation of CDK9 (Baumli et al., 2008). However, based on the results, mp3, mp12, mp26 and mp29 displayed interactions with Arg172, potentially disrupting the formation of this hydrogen bonding network (Table 4). Furthermore, Table 4 demonstrates that all mutant peptides exhibit a more negative HADDOCK score compared to temp2. The screening and identification of potentially effective mutant peptides were conducted considering multiple factors: (i) the count of peptide residues involved in hydrogen bond formation, (ii) the count of mutated positions capable of forming hydrogen bonds, (iii) the interactions between peptide residues and active site residues of CDK9, and (iv) the HADDOCK score. Based on these criteria, three peptides, namely, mp3, mp20, and mp29, were selected as potentially effective candidates.

**TABLE 4:** Sequence comparison of temp2 with six potential mutant peptides, indicating docking scores, and identifying interacting residues in the CDK9-peptide complexes using Discovery Studio Visualizer.

Name	Sequence^a^	Z-score	HADDOCK score	Interacting residues^b^	
				CDK9	Peptide
temp2	LAAKVEGQPKKLE	−2.8	−78.7	**Met1**, Ala2, Lys3, **Cys10**, **Phe12**, **Cys13**, **Glu57**, **Phe59**, Ile61, Leu64, Lys68, **Gln71**	**Ala91**, Ala92, **Lys93**, **Val94**, **Glu95**, **Lys99**, **Lys100**, Leu101, Glu102
mp3	R AA D VEGQ R K RR E	−2.6	−101.6	Met1, **Ala2**, Lys3, **Cys10**, Phe12, Cys13, **Glu57**, **Phe59**, Leu64, Arg65, Lys68, **Gln71**, **Ile82**, **Ile84**, Arg172, **Ala173**	**Arg90**, Ala91, Ala92, **Asp93**, Val94, **Glu95**, **Lys99**, **Arg100**, Arg101, **Glu102**
mp7	R AA W VEGQ R K RR E	−2.1	−92.8	**Met1**, Ala2, **Lys3**, **Cys10**, **Phe12**, Cys13, **Glu57**, **Phe59**, Leu64, Lys68, Gln71, **Ile82**	**Arg90**, **Ala91**, Val94, **Glu95**, **Lys99**, **Arg100**, Glu102
mp12	R AA D VEGQ R K DR E	−1.6	−91.5	**Met1**, Lys3, **Cys10**, Phe12, Phe59, **Leu64**, Ile67, **Lys68**, **Gln71**, Ile84, Arg172	Ala91, **Ala92**, **Asp93**, Val94, **Glu95**, **Gln97**, Lys99, **Asp100**, **Arg101**, **Glu102**
mp20	R AA T VEGQ R K RR E	−2.3	−85.4	**Met1**, **Gln4**, **Glu9**, **Phe12**, **Gly58**, Phe59, Ile67, **Gln71**, Ile84, **Thr87**, **Lys88**, Arg94, **Cys95**, **Gly97**	**Arg90**, **Thr93**, **Val94**, **Gly96**, **Arg98**, **Arg100**, **Arg101**, Glu102
mp26	D AA D VEGQ R K RR E	−2.0	−89.2	Met1, **Ala2**, **Phe12**, **Cys13**, **Glu57**, **Phe59**, Leu64, Lys68, **Gln71**, **Ile84**, Arg172, **Ala173**	**Asp90**, Ala91, Ala92, **Asp93**, Val94, **Glu95**, **Arg100**, Arg101, **Glu102**
mp29	R AA D VEGQ D K RR E	−2.5	−80.9	**Met1**, Ala2, Lys3, **Cys10**, Pro11, Cys13, **Phe12**, **Glu57**, **Phe59**, Arg65, **Lys68**, **Gln71**, Ile84, Arg172	**Arg90**, **Ala91**, Ala92, Val94, **Glu95**, **Lys99**, **Arg100**, **Arg101**, Glu102

Note.

^a^ Mutated positions were highlighted in red color.

^b^ Residues involved in hydrogen bonding are shown in Bold.

Following molecular docking analysis, we investigated the molecular interaction of the three selected peptides with a target molecule closely related to CDK9 to determine their specificity. To accomplish this, we obtained the amino acid sequences of all CDKs (CDK1-CDK20) from the Uniprot database (https://www.uniprot.org/) and aligned multiple sequences using the Clustal Omega server (https://www.ebi.ac.uk/Tools/msa/clustalo/). The alignment results revealed that CDK12 is a closely related target to CDK9 (data not shown). Additionally, previous research has demonstrated the structural and sequence proximity between CDK12 and CDK9 (Bösken et al., 2014). Consequently, we docked the three desired peptides against the CDK12 target (PDB ID: 4NST) using the HADDOCK server. The docking results exhibited that mp3, mp20, and mp29 peptides, with HADDOCK scores of −82.9 (Z-score = −1.5), −83.2 (Z-score = −1.3), and −85.4 (Z-score = −1.7), respectively, were capable of interacting with CDK12. Comparing these scores, we observed that the binding of mp20 and mp29 peptides to CDK9 yielded HADDOCK scores nearly equivalent to their binding to CDK12, although their Z-scores for binding to CDK9 were considerably lower. Conversely, the HADDOCK score and Z-score resulting from the binding of mp3 peptide to CDK9 were significantly lower than those obtained for binding to CDK12 (Table 4). Notably, a study conducted by Bösken et al. (2014) reported that the interaction between cyclin K and CDK12 occurs at residue Arg773 of CDK12. According to our findings, although the desired peptides were bound to the binding site of CDK12, but none of the peptides interacted with residue Arg773, suggesting that they are not expected to be blockers for the binding of cyclin K to CDK12 (Table 5). However, experimental studies will be necessary to confirm and validate these observations.

**TABLE 5:** Assessing the docking scores and identifying interacting residues in the potentially effective mutant peptides in complex with CDK12 using Discovery Studio Visualizer.

Name	Z-score	HADDOCK score	Interacting residue	
			CDK12	Peptide
mp3	−1.5	−82.9	**Ser717**, **Asp718**, **Trp719**, Glu763, **Glu765**, **Phe767**, Ile769, Ile772, **Lys776**, **Gln797**, **Asp798**, Phe809	**Arg90**, **Ala91**, Ala92, Glu95, **Gly96**, **Arg98**, **Lys99**, **Arg100**, **Arg101**, **Glu102**
mp20	−1.3	−83.2	Asp718, **Trp719**, **Lys764**, **Glu765**, Phe767, Lys776, **Arg779**, **Gln797**, **Asp798**, Phe809	Arg90, **Thr93**, **Val94**, Glu95, Arg98, **Arg100**, **Arg101**
mp29	−1.7	−85.4	**Ser717**, Asp718, **Trp719**, **Gly720**, **Lys721**, Leu760, **Glu765**, Phe767, **Lys776**, Ile775, **Arg779**, **Arg780**, **Gln797**	**Arg90**, Asp93, Val94, **Gly96**, **Gln97**, **Asp98**, **Lys99**, **Arg100**, **Arg101**, **Glu102**

***Note:*** Residues involved in hydrogen bonding are shown in Bold.

**FIGURE 2:**
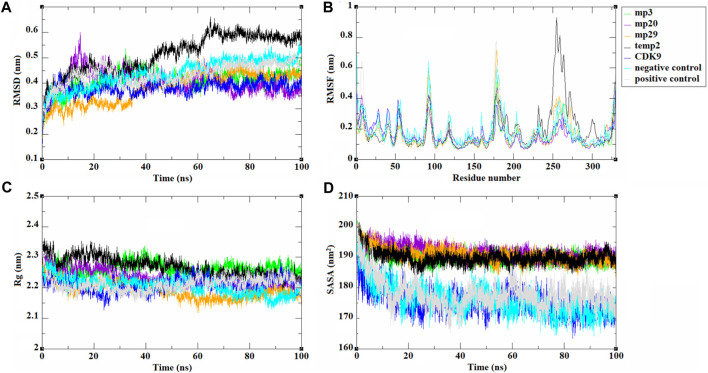
The trajectories analysis resulted from molecular dynamic simulations of selected template (temp2), three potentially effective mutant peptides (mp3, mp20, and mp29), negative and positive controls in complex with CDK9 and CDK9 alone at 310.15 K for 100 ns. The results are shown as **(A)** RMSD, **(B)** RMSF, **(C)** Rg, and **(D)** SASA plots.

Subsequently, molecular dynamics analysis was conducted to assess the stability and other behavioral characteristics of the three selected peptides (mp3, mp20, and mp29), as well as temp2, in complex with CDK9. To capture structural changes and deviations within complexes, the RMSD was utilized, which calculates the average of the entire system at each time point (Taghizadeh et al., 2022a). The resulting visual representation of these changes is depicted in Figure 2A. Comparing the RMSD values, all mutant peptides exhibited lower RMSD values than temp2 (0.511 ± 0.075 nm). Notably, mp29 (0.380 ± 0.059 nm) and mp3 (0.409 ± 0.039 nm) displayed the minimum and maximum average RMSD values, respectively (Figure 2A). These findings indicate that mp29 induces the least structural deviation, while mp3 induces the most significant conformational changes in CDK9 (Zareei et al., 2022). The complexes of mp3 and mp20 with CDK9 reached equilibrium within the initial 10 ns of the simulation and exhibited minimal fluctuations, suggesting a stable interaction. In contrast, mp29 induced a substantial conformational change in the protein after 35 ns, followed by equilibration at 60 ns. Upon binding of peptide inhibitors to the CDK9, they have the potential to induce conformational changes in the protein, thereby altering its characteristics and features (Wang et al., 2021). Moreover, the calculated average RMSD value of CDK9 was found to be 0.381 ± 0.028 nm, indicating that the stability of mp29 remained unchanged upon binding to CDK9. However, a slight increase in this value was observed after the binding of mp3 and mp20 to CDK9. Furthermore, the average RMSD values for the negative and positive controls were determined to be 0.437 ± 0.055 nm and 0.435 ± 0.043 nm, respectively (Figure 2A). To track these changes over time, an analysis of RMSF was conducted (Figure 2B). Within the complexes, notable peaks with heightened fluctuations were observed specifically in the regions of 187–210 and 232–315, known as high mobility regions. Particularly, when CDK9 was complexed with mp29, the greatest amino acid fluctuation was observed. These findings align with the results obtained from other molecular dynamics analyses (Pourmand et al., 2022), suggesting that mp29 induces higher mobility in the protein, potentially leading to partial structural instability. The analysis of Rg, which serves as an indicator of protein compactness and stability (Kianipour et al., 2022), revealed that the maximum and minimum average Rg values were in the complexes of mp3 (2.264 ± 0.019 nm) and mp29 (2.192 ± 0.025 nm) with CDK9, respectively. The Rg plots displayed slight fluctuations in the mp29, indicating the presence of small folding changes in its protein structure (Figure 2C). Additionally, the average Rg value for CDK9, as well as the negative and positive controls, was determined to be 2.211 ± 0.021 nm, 2.205 ± 0.026 nm, and 2.211 ± 0.021 nm, respectively. Notably, noticeable folding changes were observed, particularly in the negative control, as depicted in Figure 2C. SASA represents the exposed surface of a protein, considering the contact made by a hypothetical solvent sphere with the protein through van der Waals interactions. The findings revealed no noteworthy alterations in the average SASA values (SASA values of 189.899 ± 2.197, 190.037 ± 1.950, 191.727 ± 2.297, 190.681 ± 2.286 nm for temp2, mp3, mp20, and mp29, respectively), however mp29 indicates slight fluctuations in its level of openness or compactness (Figure 2D). Conversely, a notable decrease was observed in the average SASA value, accompanied by fluctuations in CDK9 (175.445 ± 3.830 nm), the negative control (176.066 ± 4.812 nm), and the positive control (177.086 ± 4.771 nm), suggesting their structural opening and compacting processes (Figure 2D).

**FIGURE 3:**
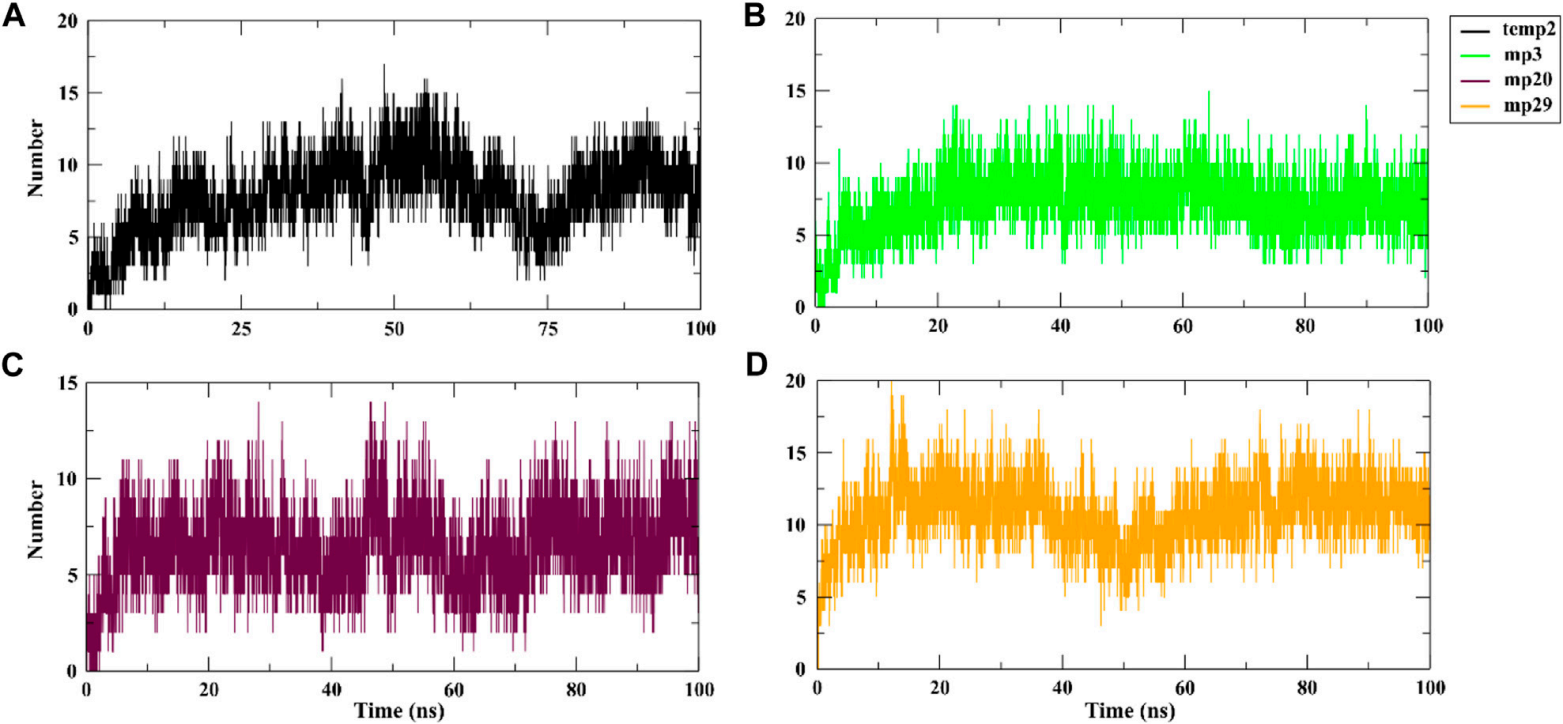
Hydrogen bond analysis of CDK9 complexed with temp2 **(A)**, mp3 **(B)**, mp20 **(C)**, and mp29 **(D)** using *gmx hbond* toolkit in gromacs after a 100 ns run at 310.15 K.

During a 100 ns simulation, we also conducted an investigation on hydrogen bonding. This is important because hydrogen bonds play a critical role in target recognition and the stability of protein-protein complexes (Hubbard and Haider, 2010). Based on the findings presented in Figure 3, the analysis reveals a clear distinction, with mp29 forming a greater number of stable hydrogen bonds, while mp3 forms more stable hydrogen bonds with CDK9, emphasizing their respective contributions to the stability of the complex. In addition, determination of protein-protein interaction energies plays a critical role in deepening our comprehension of protein-protein associations (Jiang et al., 2002). Hence, the free energy calculations provide compelling evidence for the improved binding affinity resulting from the mutation (Li et al., 2019). Analysis of the binding energies between the mutant peptides and CDK9 throughout the 100 ns simulation revealed that mp29 displayed a significantly stronger interaction with CDK9. Furthermore, this analysis demonstrated the significant contributions of van der Waals and electrostatic energies in peptide binding (Table 6). Additionally, the entropy calculation results demonstrated that the binding of temp2, mutant peptides, and positive control to CDK9 led to a decrease in entropy compared to CDK9 alone (17.364 kJ/mol) (Table 6). This decrease in entropy suggests a reduction in the flexibility of the bound state (Unarta et al., 2021). In a study conducted by Randjelović et al. (2013), multiple peptides were designed based on the structure of cyclin T1, resulting in four peptides (LQTLGFEL, FLAAKV, ESIILQ, and LQTLGF) with free binding energies ranging from −23.1 to −19.1 kcal/mol. Comparatively, our study successfully designed safe peptide inhibitors for the first time that target the CDK9-cyclin T1 complex, exhibiting both high affinity and exceptional stability.

**TABLE 6:** Calculating the energy profiles of CDK9 protein in complex with temp2, mp3, mp20, and mp29 peptides using g_mmpbsa analysis.

Peptide ID	Van der waal energy (kJ/mol)	Electrostatic energy (kJ/mol)	Polar solvation energy (kJ/mol)	SASA energy (kJ/mol)	Binding energy (kJ/mol)	Entropy (kJ/mol)
temp2	−164.770	−513.956	563.742	−23.942	−138.926	1.997
mp3	−242.831	−508.089	511.738	−34.831	−274.013	2.137
mp20	−262.964	−257.607	279.046	−34.426	−275.951	2.277
mp29	−285.311	−677.939	618.289	−38.453	−383.414	2.298
Positive control	−141.188	−403.844	317.242	−18.455	−246.245	1.085
Negative control	−96.977	−357.222	419.145	−14.931	−49.985	17.337

**FIGURE 4:**
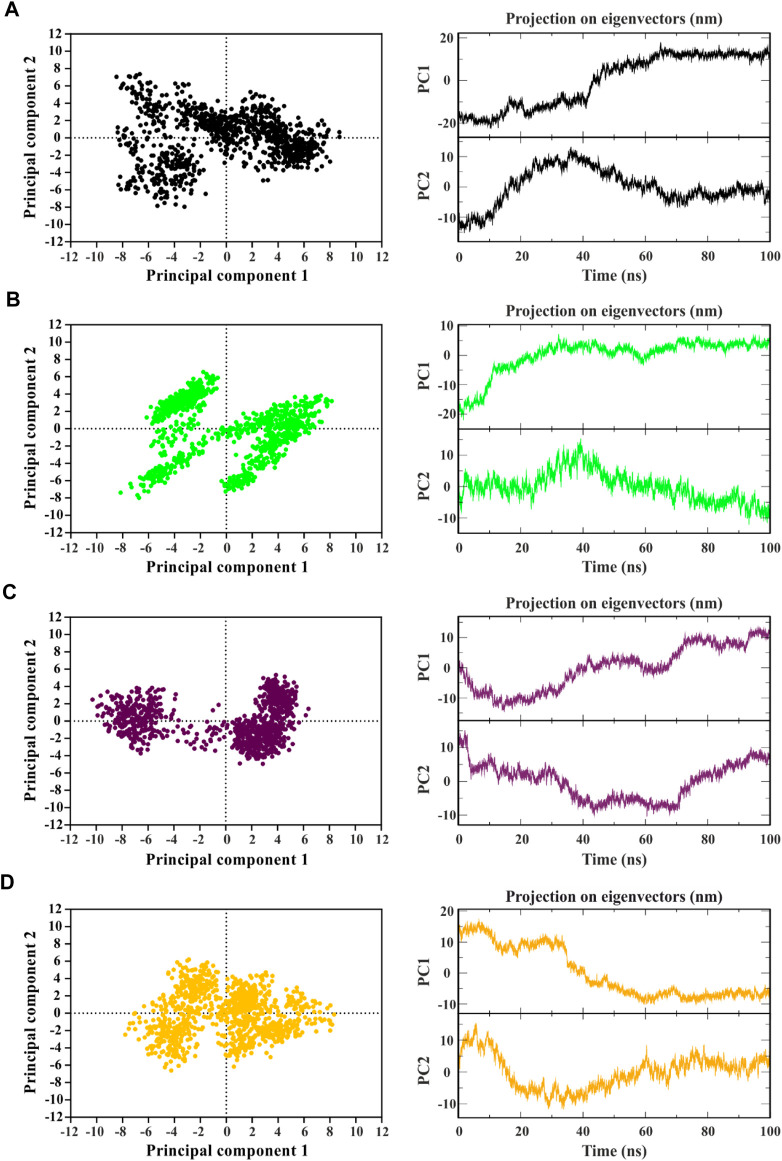
Identifying the dominant and collective motions of the protein using principal component analysis for template (temp2 **(A)**) and three potentially effective mutant peptides (mp3 **(B)**, mp20 **(C)**, and mp29 **(D)**).

The analysis of collective movements, which govern the adoption of new protein conformations, revealed that mp3 and mp20 exhibited restricted collective movements in CDK9, as they explored smaller conformational spaces compared to temp2 (Figures 4A–C). Conversely, the mp29 pattern displayed more extensive propagation across the diagram plane, indicating higher protein flexibility and increased movements in this system, covering a wider range of conformational space than temp2 (Figures 4A,D). Additionally, Figure 4 depicts the first two principal components (PCs) derived from the MD trajectories of complexes over time. Notably, there exists a noticeable correlation between PC1 and the rmsd) for both mp20-and mp29-CDK9 complexes (Figure 2A, Figures 4C,D). This correlation effectively captures the majority of the rmsd behavior, thus representing the protein’s motion throughout the entire trajectory (Maisuradze et al., 2009). While some correlation between PCs2 and rmsd can be observed during specific segments of the trajectories (e.g., 0–20 and 80–100 ns for mp20, and 0–10 ns for mp29), PC2 primarily identifies the transition from the unfolded state to the native state (Maisuradze et al., 2009). Due to these observations and the relatively minor contributions to the overall fluctuations, higher-indexed PCs are deemed less significant. Consequently, the first PC adequately represents the main characteristics of the energy landscape for both mp20-and mp29-CDK9 complexes trajectories.

In contrast, no correlation between PCs1 and the corresponding rmsd is observed in the PCs1 of the MD trajectories for both temp2-and mp3-CDK9 complexes (Figure 2A, Figures 4A, B). However, the amplitudes of fluctuation within PCs1 and PCs2 differ between temp2-and mp3-CDK9 complexes. Accordingly, the distribution of the captured fluctuations by the first PCs varies in their MD trajectories. Hence, for these trajectories, the first PC alone is insufficient to portray the primary features of the energy landscape.

**FIGURE 5:**
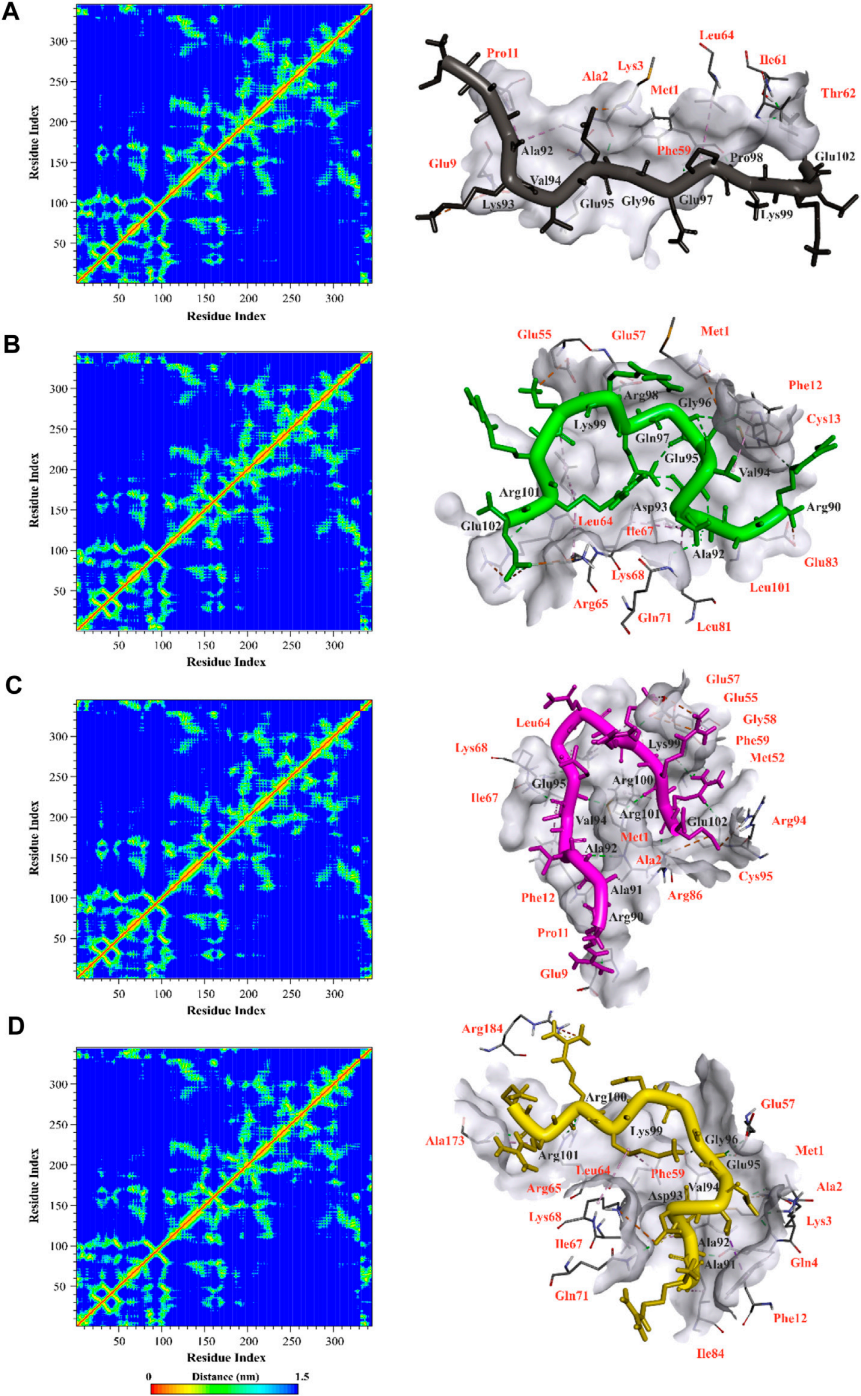
Illustration of contact map and molecular interactions of template **(A)** and mp3 **(B)**, mp20 **(C)**, and mp29 **(D)** mutant peptides in complex with CDK9 after a 100 ns molecular dynamic simulation at 310.15 K. The interacted residues of CDK9 were indicated in red color.

The distance matrices comprising the minimum distance between pairs of residues, contact map, were analyzed to observe interactions between mutated peptides and CDK9 after a 100 ns simulation. The results revealed that all mutant peptides displayed strong interactions with residues positioned within the active site of CDK9 or its surrounding region, indicating their capability to effectively mask the binding site of CDK9 and prevent cyclin T1 binding (Figure 5). Furthermore, Figure 5 provides a clear visual representation of the 3D conformation exhibited by the peptides, effectively occluding the binding site of cyclin T1 on CDK9. Accordingly, in the case of temp2, residues Ala92, Asp93, Val94, Glu95, Gly96, Gln97, Arg98, Lys99, and Glu102 were found to interact with residues Met1, Ala2, Lys3, Glu9, Pro11, Phe59, Ile61, Thr62, and Leu64 of CDK9 (Figure 5A). Similarly, for mp3, residues Arg90, Ala92, Asp93, Val94, Glu95, Gly96, Gln97, Arg98, Lys99, Arg101, and Glu102 interacted with residues Met1, Phe12, Cys13, Glu55, Glu57, Leu64, Arg65, Ile67, Lys68, Gln71, Leu81, Glu83, and Leu101 of CDK9 (Figure 5B). Additionally, in the case of mp20, residues Arg90, Ala91, Ala92, Val94, Glu95, Lys99, Arg100, Arg101, and Glu102 exhibited interactions with residues Met1, Ala2, Glu9, Pro11, Phe12, Met52, Glu55, Glu57, Gly58, Phe59, Leu64, Ile67, Lys68, Arg86, Arg94, and Cys95 of CDK9 (Figure 5C). Lastly, in the case of mp29, residues Ala91, Ala92, Asp93, Val94, Glu95, Gly96, Lys99, Arg100, and Arg101 were observed to interact with residues Met1, Ala2, Lys3, Gln4, Phe12, Glu57, Phe59, Leu64, Arg65, Ile67, Lys68, Gln71, Ile84, Ala173, and Arg184 of CDK9 (Figure 5D). The obtained results provide confirmation of the molecular docking outcomes. The N-terminal region of CDK9, including residues 16 to 108, is composed of five β structures (β1-5) and one α helix (αC) (Baumli et al., 2008). Significantly, the interaction between CDK9 and cyclin T1 is mainly helped by the αC helix, which contains the highly conserved sequence of PITALRE (Baumli et al., 2008). Based on the findings, the designed peptides have exhibited an interaction with CDK9 within this specific region.

The findings from our study indicated that there were no notable alterations observed in the different types of secondary structures throughout the duration of the simulation (Table 7), which aligns with the information provided by the Definition of Secondary Structure of Protein (DSSP) regarding the frequency of each secondary structure in the protein’s conformation (Abraham et al., 2015). Our study has found that cell-penetrating peptides are rich in arginine (Peptide sequences presented in Table 3), which aligns with our research goals. Previous studies indicated that arginine-rich peptides have a positive charge, which allows them to interact strongly with negatively charged drug molecules and cell membranes through non-covalent mechanisms, specifically electrostatic interactions (Chavda et al., 2022). The peptides we designed in our study provide further evidence of this phenomenon, which validates our findings.

**TABLE 7:** Comparison of secondary structure frequencies in CDK9 complexed with three potential mutant peptides *versus* the temp2.

Name	3-Helix	α-Helix	Turn	Bend	β-Bridge	β-Sheet	Coil	Structure
temp2	0.03	0.28	0.09	0.13	0.02	0.13	0.31	0.52
mp3	0.03	0.29	0.11	0.14	0.01	0.13	0.29	0.54
mp20	0.03	0.29	0.08	0.15	0.01	0.13	0.31	0.51
mp29	0.03	0.28	0.10	0.15	0.01	0.13	0.31	0.51

### *In silico* gene cloning of potentially effective peptides

**FIGURE 6:**
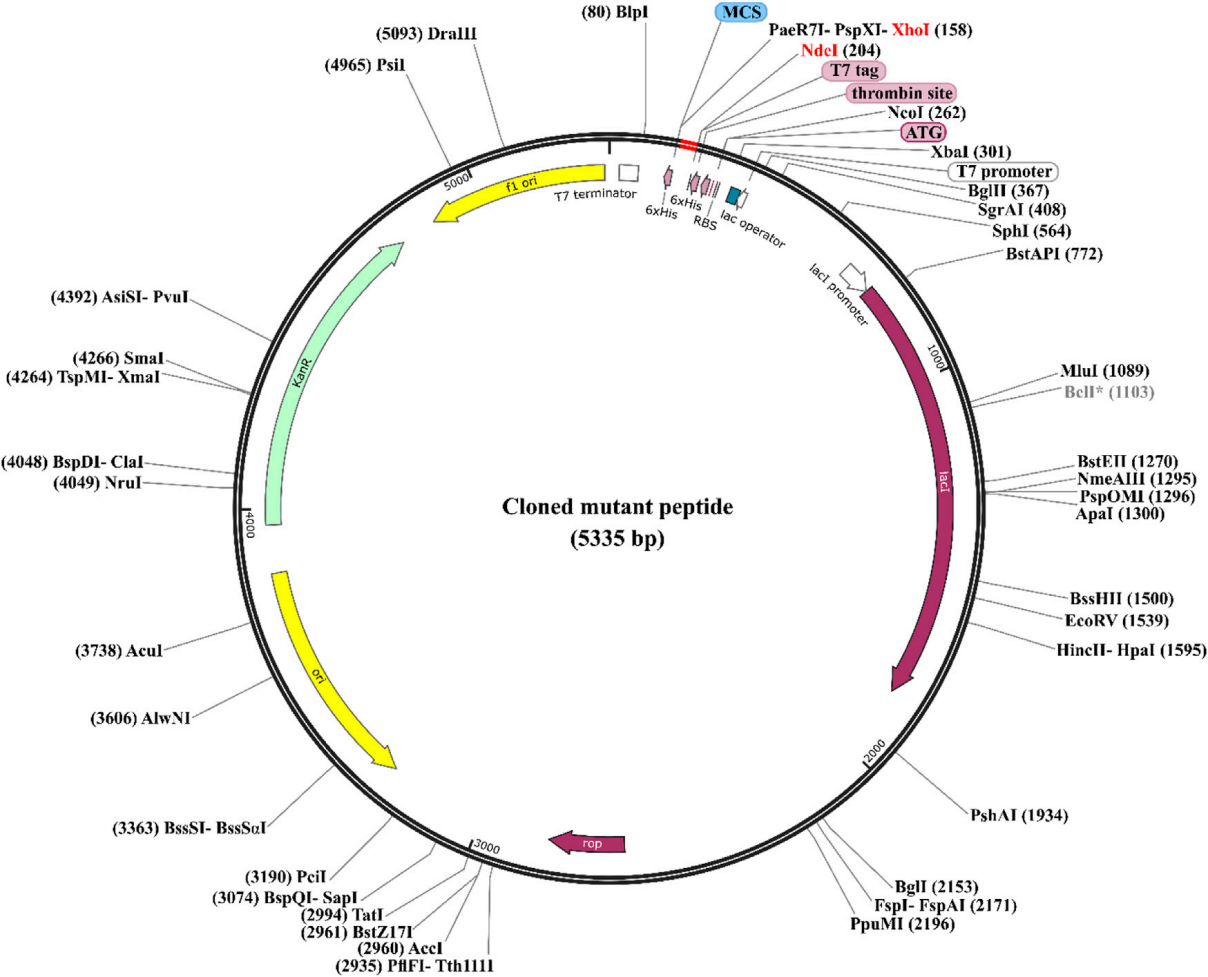
Codon Optimization, cloning, and vector construction for three potentially effective mutant peptides. The three potentially effective mutant peptides (highlighted in red) were codon optimized and cloned into the *E. coli* pET28a (+) expression vector (represented in black), in which the DNA of the mutant peptides was inserted between *XhoI* (158) and *NdeI* (204) restriction sites. To see more details of sequences, refer to the Supplementary Material S1.

We utilized *in silico* cloning techniques to express the designed peptides in a suitable host organism as part of our research. *E. coli* strain K12 was chosen as it is a widely recognized and extensively utilized expression platform, known for its established track record as a reliable cell factory (Selas Castiñeiras et al., 2018). The optimized codon sequences for all the designed peptides (mp3, mp20, and mp29) consisted of 39 base pairs, with a GC content of 53.84% and a CAI value of 1.0. To facilitate the cloning process, we introduced *XhoI* and *NdeI* restriction sites into the plasmid vector pET28a (+) using SnapGene software. The engineered peptide clone’s resulting circular map is presented in Figure 6 (refer to Supplementary Material S1 for more information). Following successful insertion, the total length of the expression vector, including the peptide DNA, measured 5,335 bp.

Solubility is crucial in recombinant protein production as it enables functional characterization, high-yield production, proper folding, and stability, as well as facilitates downstream applications in diverse research and industrial domains (Rosano and Ceccarelli, 2014). Therefore, we predicted the solubility of the designed peptides (mp3, mp20, and mp29) upon overexpression using the SOLpro server. The results showed solubility scores of 0.99, 0.96, and 0.99, respectively, indicating that they are soluble in nature upon overexpression.

## Conclusion

Based on our study where we designed and characterized a library of mutant peptides, we were able to identify three potential inhibitors (mp3, mp20, and mp29) that can preserve the formation of the CDK9-Cyclin T1 complex. These peptides have demonstrated favorable safety profiles and high affinity for the target complex. They also showed promising dynamic behavior and the ability to penetrate cells, suggesting their potential for intracellular activities. Our findings also revealed that mp3 and mp29 peptides interacted with a conserved sequence in CDK9. Successful cloning of these peptides into the *E. coli* pET28a (+) vector facilitated their recombinant production. Predictions indicated good solubility upon overexpression, which supports their suitability for downstream applications. Overall, our results highlight the potential of the designed peptides as high-affinity CDK9 blockers. Further experimental studies are necessary to validate their biological activity and assess their selectivity, although these results highlight the potential of the designed peptides as high-affinity CDK9 blockers. Ultimately, these findings contribute to the ongoing efforts in developing novel strategies for CDK9 inhibition and hold promise for potential therapeutic applications in diseases associated with dysregulated CDK9 activity.

## Data Availability

The raw data supporting the conclusions of this article will be made available by the authors, without undue reservation.
