# Supplementary material for: Structure-guided design and cloning of peptide inhibitors targeting CDK9/cyclin T1 protein-protein interaction
Source: Front Pharmacol. 2024 May 14;15:1327820. doi: 10.3389/fphar.2024.1327820 (PMC11130503; doi:10.3389/fphar.2024.1327820)
Supplement: Supplementary file 1 [file DataSheet1.zip › Supplemetary files/Supplementary Material S2.pdf]

```
import osprey
import re
import csv

osprey.start()

parallelism = osprey.Parallelism(cpuCores=16)

#defenition replace_AA:
AA=['ARG','TRP','SER','ASP','ILE','THR']

#calculates
for i in range(0,len(AA)):
    for j in range(0,len(AA)):
        for k in range(0,len(AA)):
            for l in range(0,len(AA)):
                for m in range(0,len(AA)):
                      # define a strand
                      strand = osprey.Strand('temp2_addH.pdb')
                      strand.flexibility['B90'].setLibraryRotamers(AA[i])
                      strand.flexibility['B93'].setLibraryRotamers(AA[j])
                      strand.flexibility['B98'].setLibraryRotamers(AA[k])
                      strand.flexibility['B100'].setLibraryRotamers(AA[l])
                      strand.flexibility['B101'].setLibraryRotamers(AA[m])

                      # make the conf space
                      confSpace = osprey.ConfSpace(strand)
```

```
            # choose a forcefield
            ffparams = osprey.ForcefieldParams()

            # how should we compute energies of molecules?
            ecalc = osprey.EnergyCalculator(confSpace, ffparams,parallelism=parallelism)

            # how should we define energies of conformations?
            confEcalc = osprey.ConfEnergyCalculator(confSpace, ecalc)

            # how should confs be ordered and searched?
            emat = osprey.EnergyMatrix(confEcalc)
            astar = osprey.AStarMPLP(emat, confSpace)

            # find the best sequence and rotamers
            gmec = osprey.GMECFinder(astar, confEcalc).find()

            # write the rigid GMEC to a pdb
            gmecStructure = confSpace.makeMolecule(gmec.getAssignments())
            osprey.writePdb(gmecStructure,
"temp2"+'_'+AA[i]+'_'+AA[j]+'_'+AA[k]+'_'+AA[l]+'_'+AA[m]+".pdb")


with open('log.txt', 'r') as f:
    # Create a new CSV file and write a header row
    with open('output.csv', 'w', newline='') as csvfile:
        writer = csv.writer(csvfile)
        writer.writerow(['Name', 'Energy'])

        # Loop over each line in the text file
```

```
for line in f:
    # Use a regular expression to find names ending in .pdb
    match = re.search(r'(\w+)\.pdb$', line)
    if match:
        name = match.group(1)

        writer.writerow([name, ''])

    # Use a regular expression to find numbers followed by "Energy"
    match = re.search(r'Energy\s+(-?\d+\.\d+)', line)
    if match:
        # If a match is found, add the number to the second column
        energy = match.group(1)
        writer.writerow(['', energy])
```
